# Mapping Cortico-Striatal Connectivity onto the Cortical Surface: A New Tractography-Based Approach to Study Huntington Disease

**DOI:** 10.1371/journal.pone.0053135

**Published:** 2013-02-06

**Authors:** Linda Marrakchi-Kacem, Christine Delmaire, Pamela Guevara, Fabrice Poupon, Sophie Lecomte, Alan Tucholka, Pauline Roca, Jérôme Yelnik, Alexandra Durr, Jean-François Mangin, Stéphane Lehéricy, Cyril Poupon

**Affiliations:** 1 NeuroSpin, Commissariat à l′Energie Atomique (CEA), Gif-Sur-Yvette, France; 2 Institut Fédératif de Recherche IFR49, Gif-Sur-Yvette, France; 3 Département de Neuroradiologie, Centre Hospitalier Régional Universitaire de Lille, Lille, France; 4 Centre de Neuro-Imagerie de Recherche CENIR, AP-HP, Hôpital de la Salpêtrière, Paris, France; 5 University of Concepcion, Concepcion, Chile; 6 Department of Radiology, CHUM, Notre Dame Hospital, Montreal, Canada; 7 Université Pierre et Marie Curie-Paris 6, Centre de Recherche de l′Institut du Cerveau et de la Moelle épinière, UMR-S975, Paris, France; 8 Inserm, U975, Paris, France; 9 CNRS, UMR 7225, Paris, France; 10 ICM – Institut du Cerveau et de la Moëlle épinière, Paris, France; 11 Département de Neurologie, Centre d'Investigation Clinique, AP-HP, Hôpital de la Salpêtrière, Paris, France; 12 Département de Génétique et Cytogénétique, AP-HP, Hôpital de la Salpêtrière, Paris, France; Centre Hospitalier Universitaire Vaudois Lausanne – CHUV, UNIL, Switzerland

## Abstract

Huntington disease (HD) is associated with early and severe damage to the basal ganglia and particularly the striatum. We investigated cortico-striatal connectivity modifications occurring in HD patients using a novel approach which focuses on the projection of the connectivity profile of the basal ganglia onto the cortex. This approach consists in computing, for each subcortical structure, surface connectivity measures representing its strength of connections to the cortex and comparing these measures across groups. In this study, we focused on Huntington disease as an application of this new approach. First, surface cortico-striatal connectivity measures of a group of healthy subjects were averaged in order to infer the “normal” connectivity profile of the striatum to the cortex. Second, a statistical analysis was performed from the surface connectivity measures of healthy subjects and HD patients in order to detect the cortical gyri presenting altered cortico-striatal connectivity in HD. Lastly, percentage differences of connectivity between healthy subjects and patients were inferred, for each nucleus of the striatum, from the connectivity measures of the cortical gyri presenting a significant connectivity difference between the two groups. These percentage differences characterize the axonal disruptions between the striatum and the cortex occurring in HD. We found selective region-specific degeneration of cortical connections predominating for associative and primary sensorimotor connections and with relative preservation of limbic connections. Our method can be used to infer novel connectivity-based markers of HD pathological process.

## Introduction

Huntington disease (HD) is a neurodegenerative disorder, caused by CAG repeat expansion in the HTT gene, which is located on chromosome 4 and encodes huntingtin [1]. A hallmark of HD is the progressive degeneration of the striatal medium-size spiny neurons, which represent the greatest neuronal populations of the striatum [2]. Therefore, the striatum is a part of the basal ganglia that is early and severely affected by the disease [2], [3].

Neuroimaging is increasingly used to investigate basal ganglia disorders as it can provide precise description and characterization of basal ganglia damage. While structural MRI provides some macroscopic information to characterize the atrophy of these structures, such as their volume, diffusion-weighted MRI (dMRI) can be used to characterize changes of their structure at the microscopic scale by means of rotationally invariant measures of the Brownian motion of water molecules within the tissue like the mean/ transverse/ and parallel diffusivities (MD, λ_⊥,_ λ_∥_) and the fractional anisotropy (FA) [4], [5], [6]. In addition, dMRI allows inferring their anatomical connectivity using tractography techniques [7].

In healthy volunteers, several studies relying on dMRI and tractography have analysed the connectivity of the basal ganglia [7], [8], [9], [10] as well as their main output structure, the thalamus [11]. Diffusion MRI was also used to delineate the associative, sensorimotor and limbic functional territories of the basal ganglia in healthy volunteers based on their cortical connectivity information [7], [8], [10]. Recently, this method of parcellation of the basal ganglia into functional territories was used to investigate dMRI-based measures in each territory in patients with HD [12]. However, this study did not investigate the connectivity information between the basal ganglia and the cortex or analysed connectivity measures in the cortical space. Only one study measured changes of connectivity between the striatum and the cortex in HD using tractography [13] but focusing only on the frontal cortex.

All the previous studies relying on tractography techniques (deterministic, probabilistic or bayesian) were based on tracking connections between regions of interest (ROI) which may present several limitations when studying the connections of the basal ganglia. First, they did not take into account the loops in which these structures are involved and can therefore create false direct connections between deep structures or between deep structures and the cortex. Second, they were restricted to the ROIs that were selected. Other approaches have been proposed recently, such as Tract-Based Spatial Statistics TBSS 14] which provide scalar measures including FA, MD or parallel (λ_∥_) and transverse (λ_⊥_) diffusivity on a FA-based skeleton of tracts and allows the statistical comparison of these values between groups. This method only highlights diffusion changes that are restricted to the skeleton of the main brain fibre tracts and does not provide information on the target cortical areas which connectivity is affected by the disease.

To address these limitations, we have developed a novel tractography-based approach to study the connections of the basal ganglia and the thalamus, which takes into account the known anatomy of cortico-basal ganglia loops. This approach projects the subcortico-cortical connectivity directly on the cortical surface, allowing whole brain analysis. It provides surface connectivity measures that can be used in group comparison studies to detect putative pathology-related modifications in the connections of the basal ganglia to the cortex. To illustrate this method we used it in the case of Huntington disease to investigate cortico-striatal connections modifications in HD.

The paper is organized as follows: 1) Introduction of the processing pipeline used to project the connectivity information onto the cortical surface, 2) description of the methods developed to infer the connectivity profile of each nucleus to the cortex, 3) use of the surface connectivity measures for the detection of cortical areas presenting abnormal connectivity with the striatum in HD.

## Materials and Methods

Our approach was based on the computation of surface connectivity measures characterizing the density of the connections between the striatum and the cortex. This approach used both *T*1-weighted data to extract the deep structures and the cortex, and high angular resolution diffusion-weighted data (HARDI) to recover the anatomical connectivity. The following sections describe the different steps of image processing required to compute the surface connectivity measures and to analyze them using adequate statistics.

### Structural database

Images were obtained in 15 symptomatic HD patients (8 women, 7 men, aged 46.4±6.54) and 15 age and gender matched healthy volunteers (8 women, 7 men, aged 46.4±11.76) using a Tim Trio 3T MRI system (Siemens, Erlangen). All subjects were prospectively included in the frame of a clinical project dedicated to the study of HD (Track-HD project) [3]. All patients had a genetically proven HD with an abnormal number of CAG repeats ranging from 39 to 47. Clinical characteristics of HD patients are shown in Table 1. All subjects signed an informed consent and the study was approved by the Local Ethical Committee (CPP Ile de France 6). Data were acquired using the following sequence parameters: sagittal 3D MPRAGE T1-weighted: FOV = 256×256 mm^2^, matrix 256*256, Flip angle  = 10° TE/TR = 2.98 ms/2.3 s, slice thickness TH = 1.1 mm, inversion time TI = 900 ms, 160 slices per slab, read bandwidth RBW = 240Hz/pixel; Single-shot twice refocused spin-echo diffusion-weighted (DW)-EPI: FOV = 256×256 mm^2^, TH = 2 mm, matrix 128*128, TE/TR = 86ms/12 s, GRAPPA 2, partial Fourier factor 6/8, 80 slices, RBW  = 1630 Hz/pixel, b-value = 1000 s/mm2, 50 non collinear directions uniformly distributed; Dual-echo 2D gradient field map: FOV = 176 224mm^2^, matrix 64*64, TE1/TE2  = 4.92/7.3, TH  = 3.5 mm, TR  = 0.5 s, 37 slices, RBW  = 200 Hz/pixel, flip angle  = 60°.

**Table 1:** Clinical characteristics of the subjects.

	HD patients	Healthy volunteers
Gender (M/F)	7/8	7/8
Age (years)	46.4+−6.54	46.4+−11.76
CAG	43.47+−1.64	-
Burden	357.9+−51.38	-

### T1-weighted data analysis

#### Deep nuclei segmentation

In order to investigate cortico-striatal connectivity profiles, we segmented not only the striatum but also other deep nuclei that are involved in cortico-subcortical loops. These nuclei were used in the cortico-striatal tracts selection step. Segmentation of the deep nuclei was performed from *T*1-weighted data using a deformable model with regions evolving in competition with topology constraints described in [15]. The automatic segmentation included three basal ganglia nuclei in each hemisphere (the left and right caudate nucleus (LCd and RCd, respectively), the left and right putamen (LPu and RPu), the left and right globus pallidus (LGP and RGP), and the thalamus, the main output structure of the basal ganglia (left and right thalamus: LTh and RTh, respectively). All the automatic segmentations were checked by an expert (CD) and corrected manually accordingly.

#### Cortex segmentation and parcellation

FreeSurfer v5.0 was used to extract the surface of the cortex for all the subjects [16]. A spherical resampling was performed as proposed by [17] in order to have the same number of vertices for all the subjects and a direct correspondence between these vertices across subjects. This property allowed accurate and direct matching of the surfaces of different subjects. In addition, to represent the information stemming from different subjects, an average surface was computed for each population from the individual surfaces extracted for all the subjects. The cortex of each subject was then subdivided into regions of interest using the FreeSurfer gyrus segmentation [18]. These regions were used for the computation of local connectivity statistics across subjects.

#### Tissues and CSF extraction

The gray matter GM, white matter WM and cerebrospinal fluid CSF were extracted using SPM5 software. We computed the intracranial volume of each subject by summing the volumes of GM, WM and CSF. This volume was used to normalize connectivity measures in the prospect of performing group comparison.

### Diffusion data analysis

#### Artifact removal and registration

Diffusion-weighted data were corrected from artefacts as follows. Slice outliers due to spikes or motion of the subject during the acquisition of the k-space were detected and corrected using the technique described in [19] and implemented in the BrainVISA/Connectomist-2.0 diffusion toolbox. Distortions stemming from susceptibility effects were estimated using the field map acquired together with the DW data and were also corrected using the BrainVISA/Connectomist-2.0 diffusion toolbox. Motions occurring during the acquisition were corrected using an affine registration, matching any DW data to the reference T2-weighted data acquired at b = 0*s*/*mm*2. The diffusion-weighted directions were modified accordingly. Last, the corrected DW data were matched to the *T*1-weighted data using a rigid 3D transform estimated by an automatic registration algorithm based on mutual information.

#### Tractography

To recover the anatomical connectivity of each subject, a tractography technique was employed. First, a robust mask of the brain was built from the *T*1-weighted data as described in [20]. The advantage of such a mask compared to the classical FA-thresholding based masks, is that it is based on the anatomy of each subject and is not altered by FA modifications due to a given pathology. In fact if a classical FA-thresholding mask was used, some fiber pathways could be missed by the tractography algorithm because they are not covered by the mask due to Huntington's disease related FA modifications in the voxels belonging to these pathways.

Then, the analytical Q-ball model as described in [21] was used to estimate the local underlying orientation distribution function (ODF) using a spherical harmonics order 6 and a regularization factor equal to 0.006. Finally, a streamline probabilistic tractography algorithm [22] adapted to the q-ball representation and available in BrainVISA/Connectomist-2.0 diffusion toolbox was employed. This algorithm used regularized particle trajectories and was validated using a crossing phantom composed of haemodialysis fibers [22]. For each voxel of the mask, 27 probabilistic streamlines were processed. At each step of the streamlining, the most likely direction was determined from the ODF and a random direction was chosen in a cone of aperture 60 around the direction of maximum diffusion within this cone as described in [22].

#### Selection of the cortico-striatal tracts

Connections between the cortex and the basal ganglia plus the thalamus were organized into specific circuits, according to [23]. According to this model, the striatum receives afferents from the cerebral cortex. Most areas of the neocortex except the primary visual and auditory areas have projections onto the striatum. The striatum projects to the external and internal segments of the globus pallidus and the internal globus pallidus projects in turn to the thalamus. The thalamus sends efferents to the cerebral cortex. Recent anatomical investigations have revealed a more complex organization [24].

**Figure 1:**
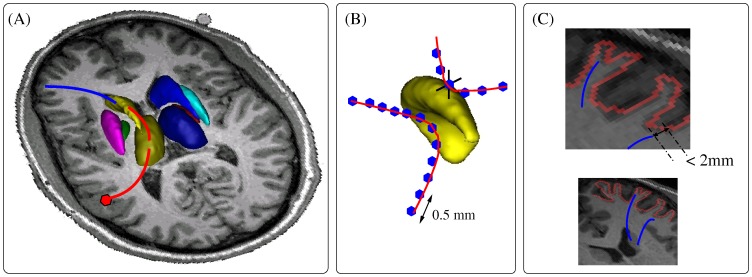
Cortico-striatal tracts selection. (A) If all basal ganglia were not taken into account in the selection process, direct connections (blue segment) but also indirect connections (red segment) of the striatum to the cortex would be studied. (B) A tract was considered as intersecting a nucleus if at least 2 points of the tract intersected the nucleus. (C) A tract was considered as intersecting the cortical surface if its distance to the surface was less than 2 mm.

If the fibre tracts linking the striatum to the cortical surface are selected from the whole brain tractogram without taking into account the segmentation of other structures like the globus pallidus or the thalamus, indirect connections between the striatum and the cortex would be included (see blue segments in Figure 1.A), leading to false positives.

We developed a selection process that took into account the segmentation of the cortical surface and of several subcortical nuclei (caudate nucleus, putamen, globus pallidus and thalamus). For each tract belonging to the whole brain tractogram obtained after the tractography step, the intersection with each subcortical nucleus and with the cortical surface was computed. The tract was considered as intersecting the nucleus if at least a segment of minimum length of the tract intersects the nucleus (to avoid tangent fibres, see Figure 1.B) and as intersecting the cortical surface if its distance to the surface is less than a given distance (2.0 mm in our case), which corresponds to the resolution of the DW data (Figure 1.C). This distance is considered to take into account the misregistration due to putative imperfect artefact removal.

The tracts were first resampled with a 0.5 mm step which corresponds to half the resolution of T1-weighted images. This selection process allowed discarding the tracts that do not intersect both the striatum and the cortical surface.

As for the tracts intersecting both the striatum and the cortical surface, two cases were obtained: either the tract links the striatum nucleus directly to the cortex (see blue segment in Figure 1.A) in which case the tract is kept, or the tract links the striatum nucleus to the cortex through an intermediate nucleus (see red segment in Figure 1.A) in which case the tract is discarded.

Through this careful selection process, all false positive tracts entailing connectivity measures are removed leading to clean subcortico-cortical tractograms.

### Surface cortico-striatal connectivity measures

The ultimate goal of this work was to compare the connectivity profiles of the striatum to the cortex between different groups and to detect a pathology related connectivity change. To this aim, we computed connectivity matrices to evaluate for each subject the number of tracts linking each vertex of the cortical surface to each nucleus of the striatum. From such connectivity matrices, we inferred for a given population P, the connectivity profile of each nucleus to each region of the cortical surface.

#### Cortico-striatal connectivity matrix

For each subject s, the number of tracts connecting each nucleus *n* to each region of the cortex was obtained by computing the intersection between the cortical surface and the fibre tracts linking n to it and obtained following the procedure described in 2.3.3. The values related to each nucleus *n* were stored in a line of a sparse connectivity matrix [25].

**Figure 2:**
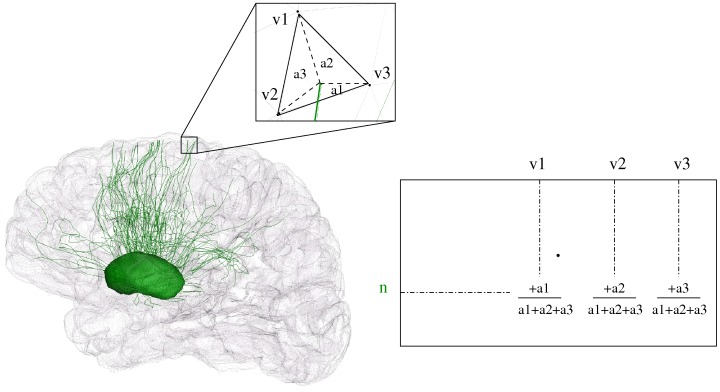
Connectivity matrix of a nucleus n to the cortex surface. v1, v2 and v3 represent the vertices of a triangle which is intersected by a fibre tract coming from n.

For each tract linking a nucleus *n* to a triangle of the cortex surface, the intersection point of the triangle and the tract (or the projection of the closest point belonging to the tract on the triangle) divides the triangle into 3 small triangles corresponding to the areas *a*_1_, *a*_2_ and *a*_3_ (Figure 2). For each vertex 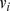, 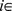
{1, 2, 3} of the triangle, the weight 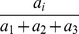 was added to the vertex position corresponding to *v_i_* in the connectivity matrix in order to provide a higher weighting to the closest vertex to the projection point. When all the tracts are processed, a connectivity matrix containing the values of connectivity 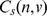 of each nucleus *n* to each vertex position *v* of the cortex is obtained. For each vertex *v* and each nucleus *n* the connectivity value 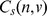 provides an estimation of the number of tracts linking *n* to *v*.

#### Cortico-striatal connectivity profile

Before comparing connectivity measures across groups it is important to define some cortical regions of interest. We focused on the gyri obtained from the FreeSurfer parcellation described in 2.4. This parcellation consisted of 34 cortical regions in each hemisphere.. To restrict for each nucleus the number of cortical regions to be analyzed, we extracted for each nucleus its connectivity profile to the cortex which means the gyri to which it is the most connected. This connectivity profile was inferred from the connectivity measures contained in the connectivity matrix of each healthy subject. For each subject s, we computed the surface connectivity measure 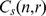 representing the number of tracts linking a nucleus n to a gyrus r.
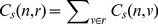
(1)


We also computed for each subject the surface 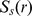 of each gyrus. The value 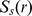 was obtained by summing the areas of the triangles of the cortical mesh belonging to the gyrus r.

For each subject, we normalized each connectivity measure 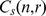 by the surface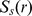. We then averaged these values across the subjects belonging to the population H of healthy subjects to obtain average connectivity measures per surface unit:
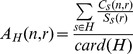
(2)


For each nucleus n, we isolated the set of gyri r for which the value 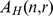 was greater than a threshold set to 1 tract per surface unit. We defined this set of gyri as the connectivity profile of the nucleus n.

### Group comparison

We compared the cortico-striatal connectivity of the two populations of healthy subjects and patients by detecting differences in surface connectivity measures. These differences were first detected using a statistical comparison and then quantified by computing a percentage difference of connectivity between the two populations. In order to take into account the intra-subject variability of surface connectivity measures we normalized the connectivity measures by the intracranial volume. Other normalization criterions were also investigated (brain or deep nuclei volumes, whole brain fibre tract number, fibre tracts crossing deep nuclei number) but were discarded because they were pathological and thus can hide pathological connectivity information if used for the normalization.

#### Statistical test

We assessed the significance of the connectivity differences between the two groups. To this aim, we computed for each subject s the normalized surface connectivity measures 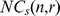 between each nucleus n and each gyrus r belonging to the connectivity profile of n:
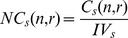
(3)Were IV*_s_* represents the intracranial volume of subject s computed as described in 2.3.3.

These normalized values were compared for the subjects of each population using a Mann-Whitney test. The choice for this statistical test was motivated by the lack of assumption about the Gaussianity of the data. For a given population P, the connectivity 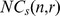 was considered as a random variable 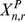 taking different values for the different subjects *s*. Using the Mann-Whitney test on the two random variables 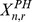 and 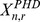 corresponding to the two populations of controls and patients respectively, we isolated for each nucleus *n* the set of gyri presenting a significant difference of connectivity, among the gyri which are part of its connectivity profile. A significance level α = 0.05 was used and a False Discovery Rate correction for multiple comparison was applied.

#### Percentage difference

For a given population P, we computed average normalized connectivity measures 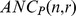 between each nucleus *n* and each cortical region of interest *r* that belongs to its connectivity profile and that presents a significant connectivity difference between the group of healthy subject and HD patients. These measures were obtained by summing up the normalized connectivity measures of all the subjects s belonging to the population P and then by dividing the sum by the number of subjects of the population (card(P)):
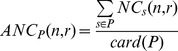
(4)


We measured the percentage difference of connectivity (PDC) between two populations, using the average normalized connectivity of healthy subjects as a reference. For a given cortical region *r* and a given nucleus *n*, the percentage difference of connectivity between the two groups of subjects was defined as follows:
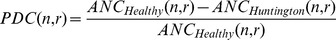
(5)where 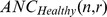 and 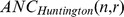 represent the average normalized number of fibre tracts connecting *n* to *r* for healthy subjects and HD patients respectively.

## Results

### Deep nuclei volumes

**Figure 3:**
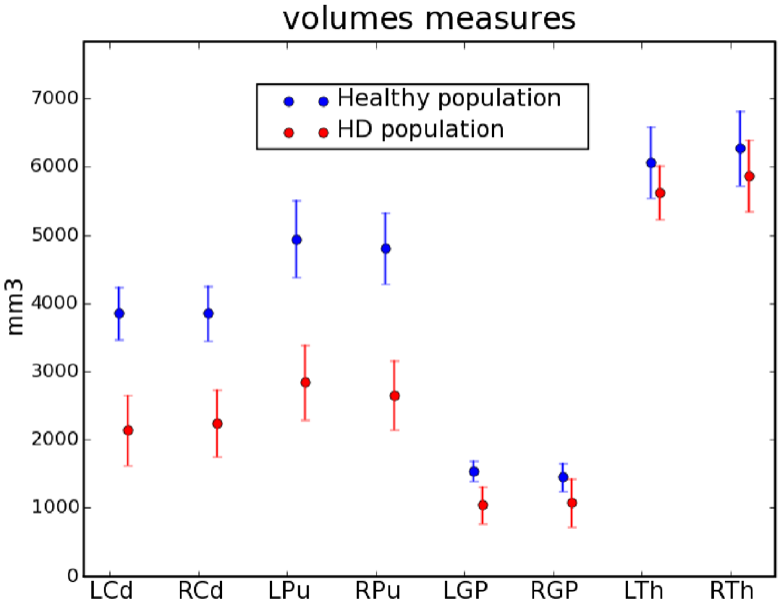
Volumes of the deep nuclei for healthy subjects and HD patients. Points represent mean values and bars standard deviations.

The volumes of the basal ganglia were measured from the manually corrected automatic segmentations. The volumes of all nuclei were systematically decreased in patients compared to controls (caudate nuclei: 43.2% and putamen: 43.6%, Figure 3).

### Streamline probabilistic tractography

**Figure 4:**
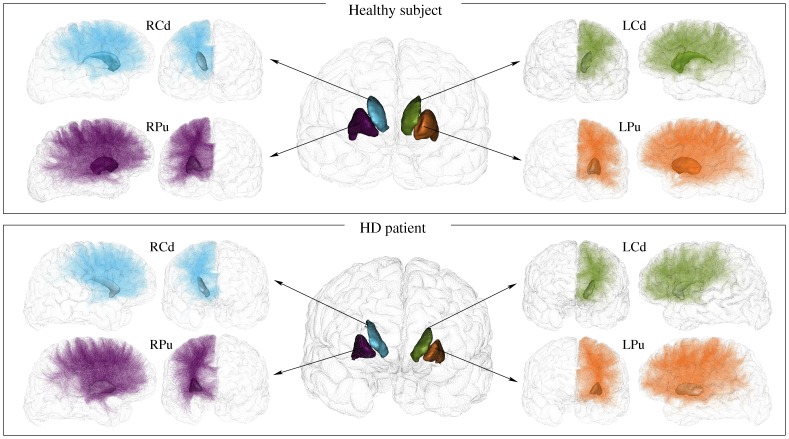
Cortico-striatal fibre tracts in one healthy subject and one HD patient. Only 1% of the actual fibre tracts were randomly selected and represented for a better rendering. In the patient, the volume of the striatum was reduced and the number of tracts obtained for each nucleus was decreased.

**Figure 5:**
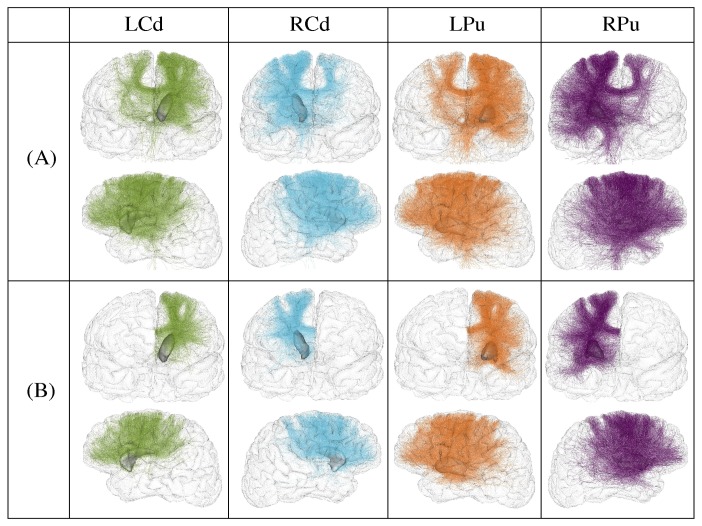
Selection of the connections of the striatum for a healthy subject. (A) selection of the tracts that intersect the striatum, (B) selection of only the direct tracts that link the striatum to the cortex. Only 1% of the actual fibre tracts were randomly selected and represented for a better rendering.

The tracts linking the striatum to the cortex were extracted from the whole set of tracts using the new selection procedure that we described in 2.3.3. An example of result of the selection of the fibre tracts linking the striatum to the cortex is provided in Figure 4 for one healthy subject and one HD patient, clearly depicting a reduced number of tracts obtained for HD patients compared to controls. In order to highlight the importance of incorporating anatomical prior knowledge in the tract selection process, we represented in Figure 5 the results of the selection of tracts passing through the striatum on a healthy subject using 2 different procedures: (A) by selecting all the tracts that intersect the striatum (a tract was considered as intersecting the striatum if at least 3 points of the tract are inside the striatum). (B) by selecting the tracts linking each nucleus of the striatum to the cortex as described in 2.3.3 and as was done in this paper. The Figure 5 clearly shows that the new selection procedure removes all the indirect tracts that connect each nucleus to the cortex and keeps only direct ones.

### Surface cortico-striatal connectivity measures

**Figure 6:**
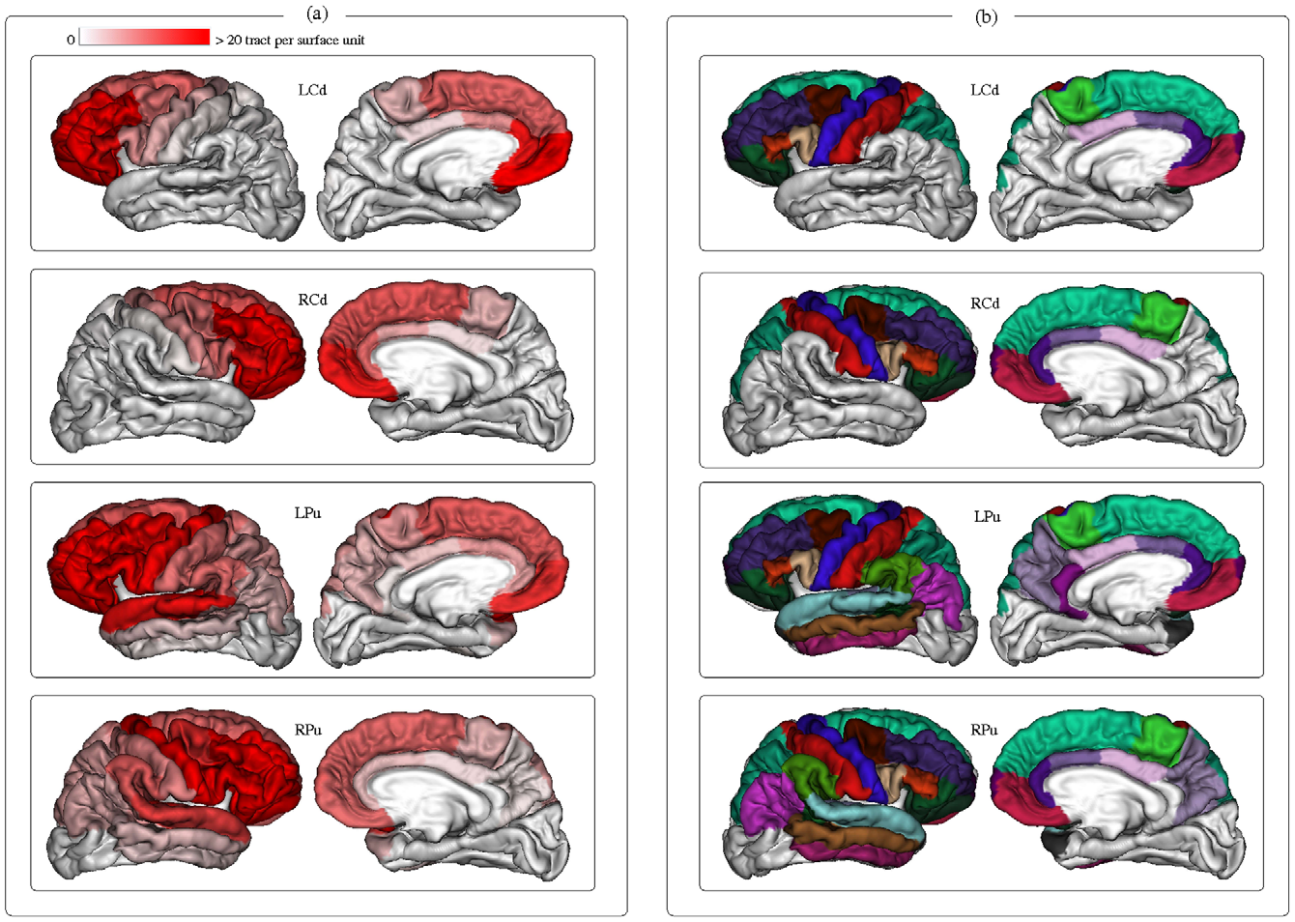
Connectivity profile of the striatum to the cortex. (A) Average connectivity measures per surface unit for each nucleus and each gyrus. (B) Cortical gyri constituting the connectivity profile of each nucleus.

Surface connectivity measures were inferred between each nucleus of the striatum (caudate nucleus and putamen) and all the cortical gyri. For each nucleus and for each gyrus, an average connectivity measure per surface unit was computed for the group of healthy subjects as described in equation (2). The values obtained for each nucleus were mapped onto an average cortical surface computed from healthy subjects, and represented using a red color gradient palette showing the strength of the connectivity to each gyrus (Figure 6(A)). The connectivity profiles of each nucleus (which means the cortical gyri having an average connectivity value greater than 1 tract per surface unit) were represented in Figure 6(B).

### Group comparison

**Figure 7:**
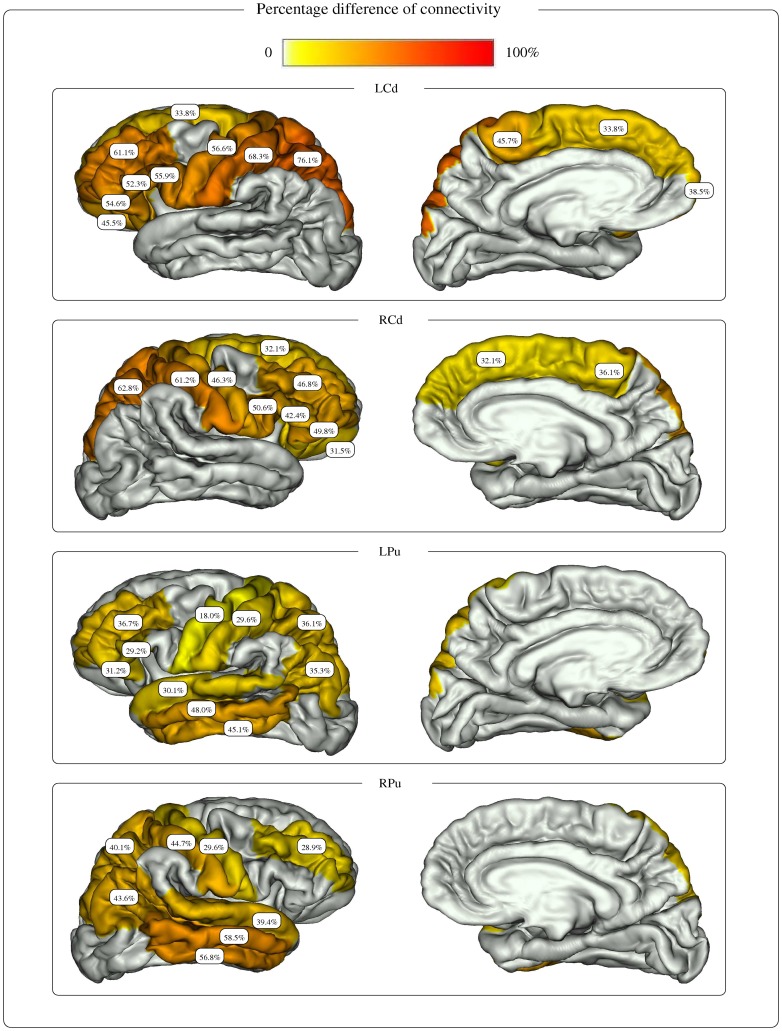
Percentage difference of connectivity for the striatum nuclei. The figure shows the percentage difference of connectivity between healthy subjects and HD patients, obtained for each nucleus of the striatum and cortical gyri belonging to the connectivity profile of the nucleus and presenting a significant connectivity difference between healthy subjects and HD patients.

Among the cortical regions constituting the cortical connectivity profile of each nucleus, the gyri presenting a significant difference of connectivity between the two populations were detected using a Mann-Whitney test (Table 2). Cortical regions associated with non significant p-values were not considered for further analysis. The percentage differences of connectivity PDC(*n*, *r*) obtained for each nucleus on the selected cortical regions are shown in Figure 7.

**Table 2:** P values obtained using a Mann-Whitney non parametric test for each nucleus on the cortical regions belonging to its connectivity profile.

Cortical region of interest	LCd	RCd	LPu	RPu
**Frontal**				
(32) Frontal Pole	**0.009552**	0.233960	0.287755	0.393731
(27) Rostral Middle Frontal	**0.000103**	**0.000653**	**0.000031**	**0.002905**
(28) Superior Frontal	**0.004221**	**0.005372**	0.475195	0.042595
(3) Caudal Middle Frontal	0.018101	0.016334	0.042595	0.186256
(12) Lateral Orbito Frontal	**0.000087**	**0.000488**	0.005372	0.006046
(14) Medial Orbito Frontal	0.491727	0.246865	0.135847	0.046492
(17) Para Central	**0.001000**	**0.007623**	0.154764	0.070447
(18) Pars Opercularis	**0.001511**	**0.000754**	0.009552	0.006795
(19) Pars Orbitalis	**0.007542**	**0.000103**	**0.001727**	0.009552
(20) Pars Triangularis	**0.000195**	**0.000653**	**0.000653**	0.006795
(24) Precentral	**0.001149**	**0.003733**	**0.003733**	**0.002905**
**Parietal**				
(22) Post Central	**0.000228**	**0.000869**	**0.001319**	**0.000087**
(29) Superior Parietal	**0.000053**	**0.000420**	**0.002905**	**0.001000**
(8) Inferior Parietal	-------------	-------------	**0.003733**	**0.000653**
(25) Precuneus	-------------	-------------	0.145098	0.022127
(31) Supra Marginal	-------------	-------------	0.164846	0.004766
**Temporal**				
(30) Superior Temporal	-------------	-------------	**0.003733**	**0.000311**
(15) Middle Temporal	-------------	-------------	**0.000267**	**0.000031**
(9) Inferior Temporal	-------------	-------------	**0.000142**	**0.000142**
(33) Temporal Pole	-------------	-------------	0.127008	0.409773
(34) Transverse Temporal	-------------	-------------	0.409772	**0.001727**
(13) Lingual	-------------	-------------	-------------	-------------
(7) Fusiform	-------------	-------------	-------------	-------------
(16) Para Hippocampal	-------------	-------------	-------------	-------------
(6) Entorhinal	-------------	-------------	-------------	-------------
**Occipital**				
(21) Peri Calcarine	-------------	-------------	-------------	-------------
(11) Lateral Occipital	-------------	-------------	-------------	-------------
(5) Cuneus	-------------	-------------	-------------	-------------
**Cingulate**				
(26) Rostral Anterior Cingulate	0.175343	0.287755	0.102921	0.393731
(2) Caudal Anterior Cingulate	0.018101	0.186256	0.102921	0.118578
(23) Posterior Cingulate	0.032462	0.377868	0.246865	0.442287
(10) Isthmus Cingulate	-------------	-------------	0.475195	-------------

The regions that did not belong to the connectivity profile of the nucleus are represented by dashes (-----). The rows correspond to the cortical regions of interest and the columns correspond to the p values of the Mann-Whitney test for the left caudate, left putamen, right caudate and right putamen respectively. The significance levels obtained with the FDR multi-comparison correction were equal to: 0.010168, 0.008319, 0.003991, and 0.004193 for the LCd, RCd, LPu and RPu respectively.The p-values that were lower than the significance level were represented in bold.

For the caudate nucleus, cortical regions depicting significantly reduced connections predominated in the parietal lobes (posterior parietal regions and primary sensory area), followed by the frontal lobes (dorsal and ventral lateral, frontopolar, lateral orbito-frontal and primary motor). For the left caudate nucleus, the reduction ranged from 33.8% for the left frontal superior gyrus to 76.1% for the left posterior parietal area. For the right caudate nucleus, the PDC values ranged from 31.5% for the right lateral orbito-frontal gyrus to 62.8% for the right posterior parietal area.

For the putamen, cortical regions with reduced connections predominated in associative temporal, the dorsal and ventral frontal areas, and parietal regions (posterior parietal and angular cortices), followed by the primary sensorimotor cortex. The PDC values for the left putamen varied from 18.0% for the left precentral gyrus to 48.0% for the left middle temporal gyrus. The PDC for the right putamen ranged from 28.9% for the right dorso lateral prefrontal gyrus to 58.5% for the right middle temporal gyrus.

## Discussion

In this work, we have developed a novel approach to infer and map the connectivity of deep brain nuclei (basal ganglia and thalamus) directly onto the cortical surface. We applied this approach to investigate cortico-striatal connectivity in HD patients and showed a selective region-specific degeneration of cortical connections of the striatum by providing quantitative measures of the percentage difference of connectivity between each nucleus and cortical area. Our method for studying connectivity between the cortex and deep brain structures differs from previous ones in healthy volunteers, which relied on tractography-based subdivision of the basal ganglia [7], [8], [9], [10] and thalamus [10], [11] based on their connectivity profile with predefined regions of the cortex. In contrast, we focused on the circuit linking the deep nuclei to the cortex and we mapped the connectivity information onto the cortical surface. In order to quantify territory-specific disconnections between the striatum and the cortex, we developed a processing pipeline working on the cortical surface. It consisted in computing surface connectivity measures of the cortico-striatal connectivity which gave access to the strength of connection between each nucleus and any cortical region, and enabled the inference of a percentage difference of connectivity between each of these nuclei and any cortical region between healthy subjects and patients. This tool efficiently provided the functional areas of the cortex presenting significant modifications of connectivity in HD patients compared with controls.

Our results confirm the selective region-specific degeneration of cortico-striatal connections in HD. First, we found a greater degeneration of associative temporal, parietal and frontal cortico-striatal connections and a relative preservation of limbic connections. There was also relatively larger reductions in primary sensory than motor connections. These results are in line with histological data in HD patient brains which showed prominent degeneration of both sensorimotor and associative parts of the striatum in late stages of HD as well as comparably less involvement of the ventral limbic striatum [2]. Using diffusion imaging, several studies have shown that the basal ganglia and cortico-striato-pallidal networks are affected by the pathological process [12], [13], [26], [27]. Using ROI approaches, higher FA and MD values have been reported in the putamen, the globus pallidus and the caudate nucleus in symptomatic HD patients [26] and in presymptomatic gene carriers [13], [27]. In the white matter, studies using voxel-based or ROI analysis have found lower FA values in frontal white matter, the corpus callosum, the internal capsule and the white matter underlying the central areas in premanifest gene carriers [27], [28], [29] and HD patients [27], [29]. Other studies investigated diffusion measures along the skeleton of tracts using TBSS [14], [30], [31]. They reported decreased FA and increased λ_∥_ and λ_⊥_ in several associative white matter fasciculi in HD patients [31] and in the corticospinal tract in premanifest gene carriers [30]. Only two studies investigated cortico-striatal connectivity using probabilistic tractography [12], [13]. Kloppel et al. (2008) have demonstrated reduced connectivity between the frontal cortex and the body of the caudate nucleus in presymptomatic gene carriers [13]. One study parcellated the striatum into subregions based on connectivity with the cerebral cortex [12]. These authors found larger diffusion changes in sensorimotor striatal subregions of the caudate nucleus and putamen, which correlated with motor symptoms. They suggested that the motor cortico-striatal circuit was selectively vulnerable in HD.

We also provided quantitative measures of the percentage difference of the connectivity between each nucleus and cortical area. For each nucleus, the percentage difference of connectivity was greater than 18% in several cortical regions including the frontal cortex. The important involvement of frontal connection was in good agreement with previous reports [13], [32]. Cortico-striatal connectivity differences also predominated in associative areas (parietal, frontal, and temporal) and in the sensorimotor cortex in accordance with several morphological studies, which reported cortical atrophy in these regions [3], [33], [34]. Regional variations in cortico-striatal changes were also observed in accordance with the heterogeneity of the cortical atrophy reported in HD [33]. In line with a previous study, we found greater involvement of the primary sensory and posterior parietal cortex [12]. However, in contrast to this study, reduction in connections did not predominate in the primary motor network, which was as affected as other frontal connections, and we observed reduced temporal connections. Differences between studies may reflect differences in the methodology or in the selection of subjects.

Overall our data suggests involvement of associative and sensorimotor connections of the striatum, with associative connections more affected than sensorimotor ones, and relative preservation of limbic connections. Reduced connections may represent the basis of altered cognitive functions particularly in domains that engage fronto-striatal circuitry (e.g., executive functions, motor and psychomotor speed) 35,36].

## Conclusions

In this paper, we introduced a novel approach for the study of the cortico-subcortical connectivity which consists in computing surface connectivity measures. Projecting the connectivity profiles onto the cortical mantel was relevant as it enabled to detect the atrophy of the cortical connections of the striatum directly in the frame of functional areas. We applied this novel approach in the frame of a clinical study of HD and we showed that it adequately and efficiently detected differences in cortico-striatal connectivity between healthy subjects and HD patients. Reduction in connectivity predominated in associative and sensorimotor regions in good agreement with the known pathophysiology of HD. In the future, this approach will be used to investigate other neurodegenerative pathologies involving the basal ganglia, and improvements will be done to provide accurate information about the affected areas, including longitudinal changes and correlation between the cortico-basal ganglia connectivity modifications and the clinical stage of patients.
